# The pan-genome of *Treponema pallidum* reveals differences in genome plasticity between subspecies related to venereal and non-venereal syphilis

**DOI:** 10.1186/s12864-019-6430-6

**Published:** 2020-01-10

**Authors:** Arun Kumar Jaiswal, Sandeep Tiwari, Syed Babar Jamal, Letícia de Castro Oliveira, Leandro Gomes Alves, Vasco Azevedo, Preetam Ghosh, Carlo Jose Freira Oliveira, Siomar C. Soares

**Affiliations:** 1 PG Program in Bioinformatics, Institute of Biological Sciences, Federal University of Minas Gerais, Belo Horizonte, MG Brazil; 2 Department of Immunology, Microbiology and Parasitology, Institute of Biological Sciences and Natural Sciences, Federal University of Triângulo Mineiro (UFTM), Uberaba, MG Brazil; 3 Department of Biological Sciences, National University of Medical Sciences, Abid Majeed Road, Rawalpindi, Punjab 46000 Pakistan; 4 Department of Computer Science, Virginia Commonwealth University, Richmond, VA-23284 USA

**Keywords:** Pan-genome, Core genome, Singletons, *Treponema pallidum*, Syphilis

## Abstract

**Background:**

Spirochetal organisms of the *Treponema* genus are responsible for causing Treponematoses. Pathogenic treponemes is a Gram-negative, motile, spirochete pathogen that causes syphilis in human. *Treponema pallidum* subsp. *endemicum* (TEN) causes endemic syphilis (bejel); *T. pallidum* subsp. *pallidum* (TPA) causes venereal syphilis; *T. pallidum* subsp. pertenue (TPE) causes yaws; and *T. pallidum* subsp. *Ccarateum* causes pinta. Out of these four high morbidity diseases, venereal syphilis is mediated by sexual contact; the other three diseases are transmitted by close personal contact. The global distribution of syphilis is alarming and there is an increasing need of proper treatment and preventive measures. Unfortunately, effective measures are limited.

**Results:**

Here, the genome sequences of 53 *T. pallidum* strains isolated from different parts of the world and a diverse range of hosts were comparatively analysed using pan-genomic strategy. Phylogenomic, pan-genomic, core genomic and singleton analysis disclosed the close connection among all strains of the pathogen *T. pallidum*, its clonal behaviour and showed increases in the sizes of the pan-genome. Based on the genome plasticity analysis of the subsets containing the subspecies *T pallidum* subsp. *pallidum*, *T. pallidum* subsp. *endemicum* and *T. pallidum* subsp. *pertenue*, we found differences in the presence/absence of pathogenicity islands (PAIs) and genomic islands (GIs) on subsp.-based study.

**Conclusions:**

In summary, we identified four pathogenicity islands (PAIs), eight genomic islands (GIs) in subsp. *pallidum*, whereas subsp. *endemicum* has three PAIs and seven GIs and subsp. *pertenue* harbours three PAIs and eight GIs. Concerning the presence of genes in PAIs and GIs, we found some genes related to lipid and amino acid biosynthesis that were only present in the subsp. of *T. pallidum,* compared to *T. pallidum* subsp. *endemicum* and *T. pallidum* subsp. *pertenue*.

## Background

Spirochetal organisms of the *Treponema* genus are responsible for causing Treponematoses. Pathogenic treponemes cause multi-stage infections like endemic syphilis, venereal syphilis, yaws and pinta. These infections have many similarities, but they can be differentiated based on epidemiological, clinical and geographical criteria [1–3]. Primarily, the pathogenic treponemes can be classified based on the clinical symptoms of the respective disease they cause. *Treponema pallidum* subsp. *endemicum* causes endemic syphilis; *T. pallidum* subsp. *pallidum* causes venereal syphilis; *T. pallidum* subsp. *pertenue* causes yaws; and *T. pallidum* subsp. *carateum* causes pinta. Out of these four high morbidity diseases, venereal syphilis is only transmitted by sexual contact; the other three diseases are transmitted by close personal contact [2].

It is estimated by the World Health Organization (WHO) that there are 12 million new cases of syphilis annually and the aggregated cases of yaws, bejel, and pinta (the endemic treponematoses) are approximately 2.5 million globally, although good surveillance data is not available. The infections caused by *T. pallidum* are characterized by periods of active clinical disease interrupted by episodes of asymptomatic latent infection and may cause life-long infections in untreated individuals [4, 5]. *Treponema pallidum* is a Gram-negative, motile, spirochete human pathogen. Syphilis is a multistage infectious disease that can be communicated between sexual partners through active lesions or from an infected woman to her fetus during pregnancy [6, 7]. Syphilis has a worldwide distribution (e.g. Africa has a high incidence), affecting every country and continent except perhaps Antarctica [8–12]. The stages of syphilis have been divided on the basis of clinical findings that lead to treatment and follow-up. Syphilis chancres may go unnoticed primarily due to their well-documented painless nature and if they are present in those parts of the body that are difficult to visualize (e.g. cervix, throat or anus/rectum) [13]. Furthermore, due to pleomorphic appearance and lack of physician familiarity with the expressions of syphilis, their lesions may be misdiagnosed. Secondary, syphilis may manifest itself through severe rashes that may go unobserved by the patient or may mimic an extensive condition [8]. *T. pallidum* is completely sensitive to penicillin treatment, despite the use of this antibiotic for seven decades in treating syphilis infections. Standard treatment of uncomplicated syphilis with parenteral Benzathine penicillin G is highly effective at all stages. Many antibiotics’ resistance (e.g Macrolide and Clindamycin resistance) has been reported in several countries [6]. The ongoing high rate of syphilis worldwide, despite the availability of inexpensive and effective treatment, presents the most convincing argument for the need of developing new and potent vaccine against syphilis [14]. Despite the WHO’s Initiative for the Global Elimination of Congenital Syphilis, an intensive syphilis-targeted public health control has been undertaken to reduce the incidence; however, it has not been achieved yet [14]. Specifically, the reasons for failure are multifactorial; some of the responsibility can be attributed to the difficulty in the diagnosis of syphilis and treatment, and lack of access or use of prenatal screening programs [15]. The advancement in the field of genomics and cost-effective sequencing technologies has transformed the human bacterial pathogens study and helped in the improvement of vaccine designing technologies. A new and emerging methodology to get deep insight of the genome of a species or genus is the pan-genomics approach, which was introduced by Tettelin and collaborators in 2005 working with *Streptococcus agalactiae* [16]. Pan-genome provides us with the complete and non-redundant collection of genes from a species or genus and is composed of three subsets (core genome, shared genome and singletons): the core genome, which is the collection of all the genes commonly shared between all the genomes used as dataset; the shared genome, which contains only the genes shared between two or more strains, which are not present in all strains of the dataset; and, the singletons, which are present only in one strain and are referred to as strain-specific genes.

The first genome of *T. pallidum* subsp. *pallidum* (strain Nichols) was sequenced in 1998. The organism has a comparatively small genome and only 55% of *T. pallidum*’s 1041 open reading frames are recognized to have a biological function, which indicates that it uses host biosynthesis to complete some of its metabolic needs [3]. The DNA-DNA hybridization studies showed homology between DNA of venereal syphilis spirochete and DNA of culturable treponemes (*T. phagedenis* and its biotypes Reiter and Kazan) was less than 5% identical, but was indistinguishable from DNA of the yaws spirochete *T. pallidum* [3, 17, 18]. This study led to the reclassification of the agents of endemic syphilis, venereal syphilis and yaws as *T. pallidum* subsp. *endemicum*, *Treponema pallidum* subsp. *pallidum* and *T. pallidum* subsp. *pertenue*, respectively. Genomic sequencing has recognized these subspecies as clonal, but forming distinct genetic clusters [2, 3].

In this work, we perform a pan-genome approach to better understand the differences of *Treponema pallidum* infections in the broad spectrum and how genome plasticity is related to the symptom patterns. For pan-genomic comparative analyses, we used 53 *T. pallidum* strains. We present phylo-genomic correlations between all *T. pallidum* strains. Furthermore, we describe the “pan-genome”, which is the complete inventory of genes found in any member of the species; the “core genome”, which is important for basic life processes; and the “singletons”, which are normally related to environmental fitness and adaptation to host. Finally, we provide insights into the specific subsets (singletons and the pan- and core genomes) of 53 genomes of *T pallidum* strains and correlate these subsets with the plasticity of pathogenicity islands and virulence genes.

## Results

### Phylogenomics study of *Treponema pallidum* strains

**Fig. 1:**
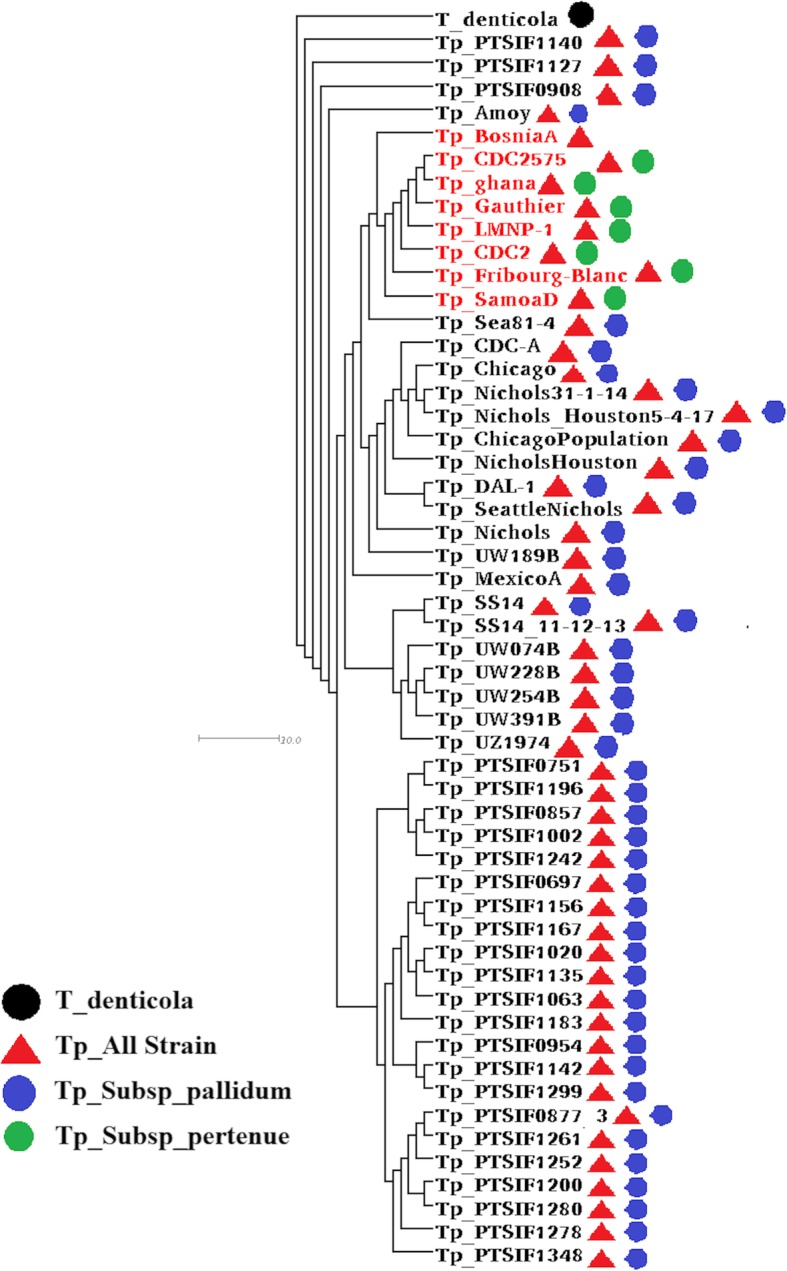
Phylogenomic tree analysis of 53 Strains of *Treponema pallidum.* The generated distance matrix data from Gegenees was used to generate a phylogenomic tree with SplitsTree (version 4.14.5) using neighbour joining method to create a dendogram. The strains name in the clade represented in red and black showed the Non-venereal and venereal strains of *Treponema pallidum,* respectively. Non-venereal *Treponema pallidum* strains are present in same clade*.* The shapes (circle and triangle) next to the name of the strain indicate the subset of strains used for Pangenome analysis according to the color of the legend respectively

The phylogenomics relationships between *T. pallidum* strains were determined using Gegenees [19]. Furthermore, all genome sequences were cross-compared to generate a phylogenomic tree and to plot a heatmap. According to the generated phylogenomic tree, closely related strains appeared in the same cluster. The subspecies responsible for non-venereal syphilis is *Treponema pallidum* subsp. *endemicum* (TEN) and *T. pallidum* subsp. *pertenue* (TPE) strains appeared in closely related clusters (Fig. 1). The *T. pallidum* subspecies strains responsible for venereal syphilis formed different clusters. Additionally, *T. pallidum* strain BosniaA (subsp. *endemicum*) was positioned between the clusters of *Treponema pallidum* subsp. *Pertenue* and venereal syphilis (*Treponema pallidum* subsp. *pallidum*)*.* According to the heatmap, the non-venereal isolates are 100% similar to each other and many of the venereal isolates are 100% similar to each other, but the two groups show some difference **(**Additional file 1: Figure S2). Moreover, the heatmap indicated the clonal-like behavior of *T. pallidum* subsp., compared with the isolates other than genital, anal or Neurosyphilitic samples, which showed similarities ranging from 97 to 100%.

### The Pan-genome, Core genome and singletons of *Treponema pallidum*

**Fig. 2:**
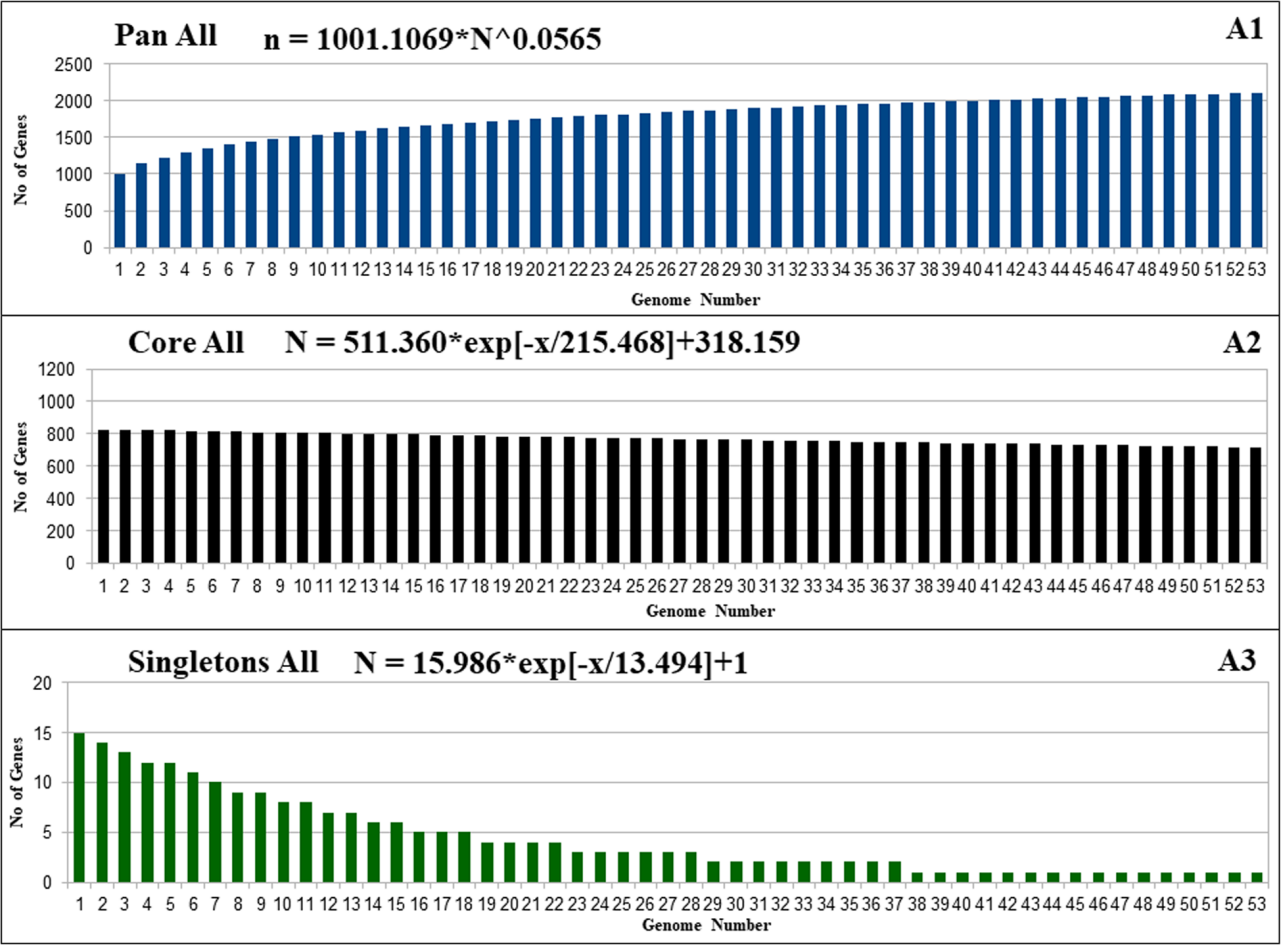
Pan-genome, core genome and singletons of *T. pallidum*. A1/A2/A3, respectively, showing the pan-genome, core genome and singletons development using 53 strains of *T. pallidum*

**Fig. 3:**
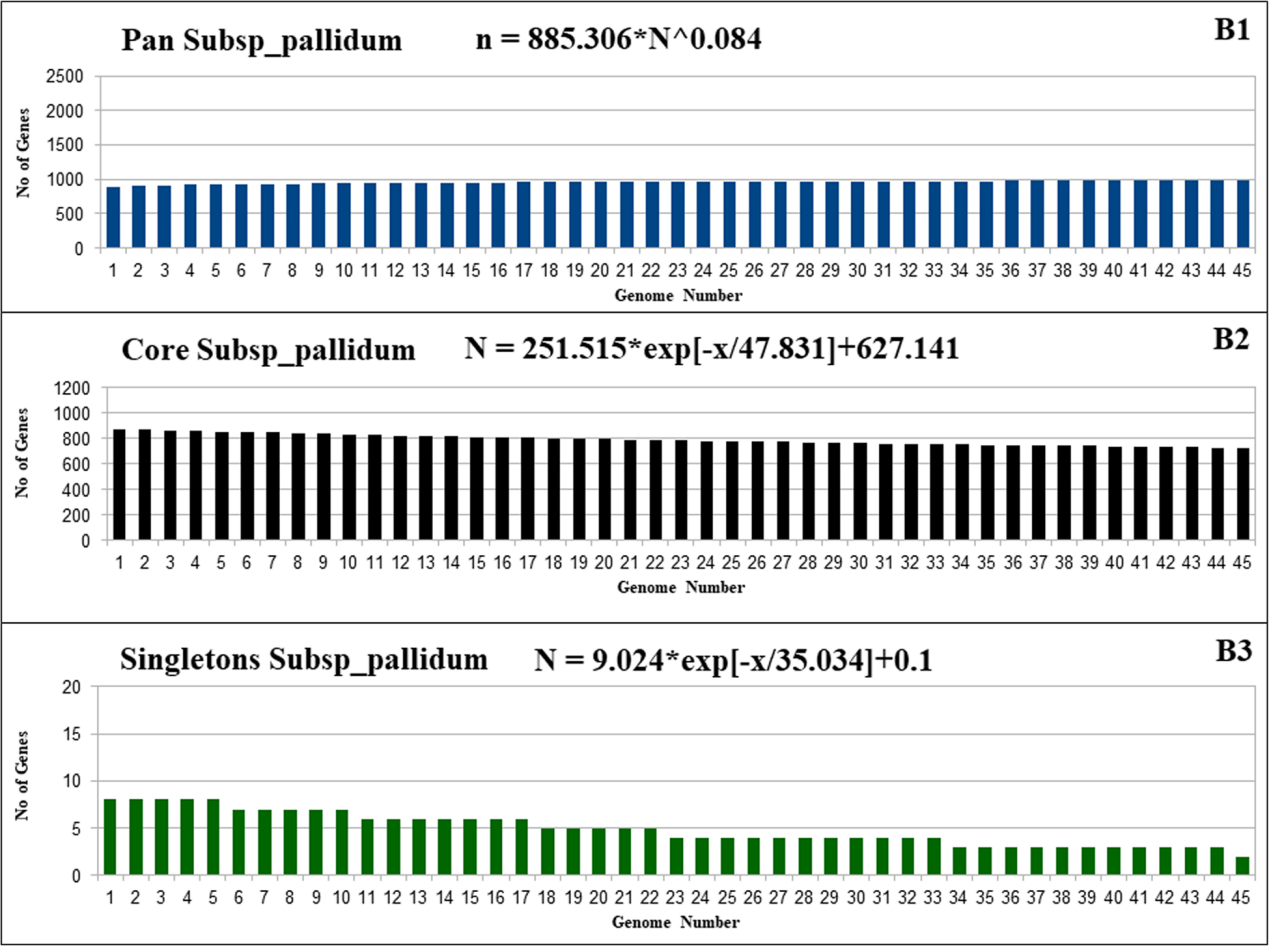
Pan-genome, core genome and singletons of *T. pallidum* Subsp_pallidum. B1/B2/B3, respectively, showing the pan-genome, core genome and singletons development using 45 strains belonging to subspecies *pallidum*

**Fig. 4:**
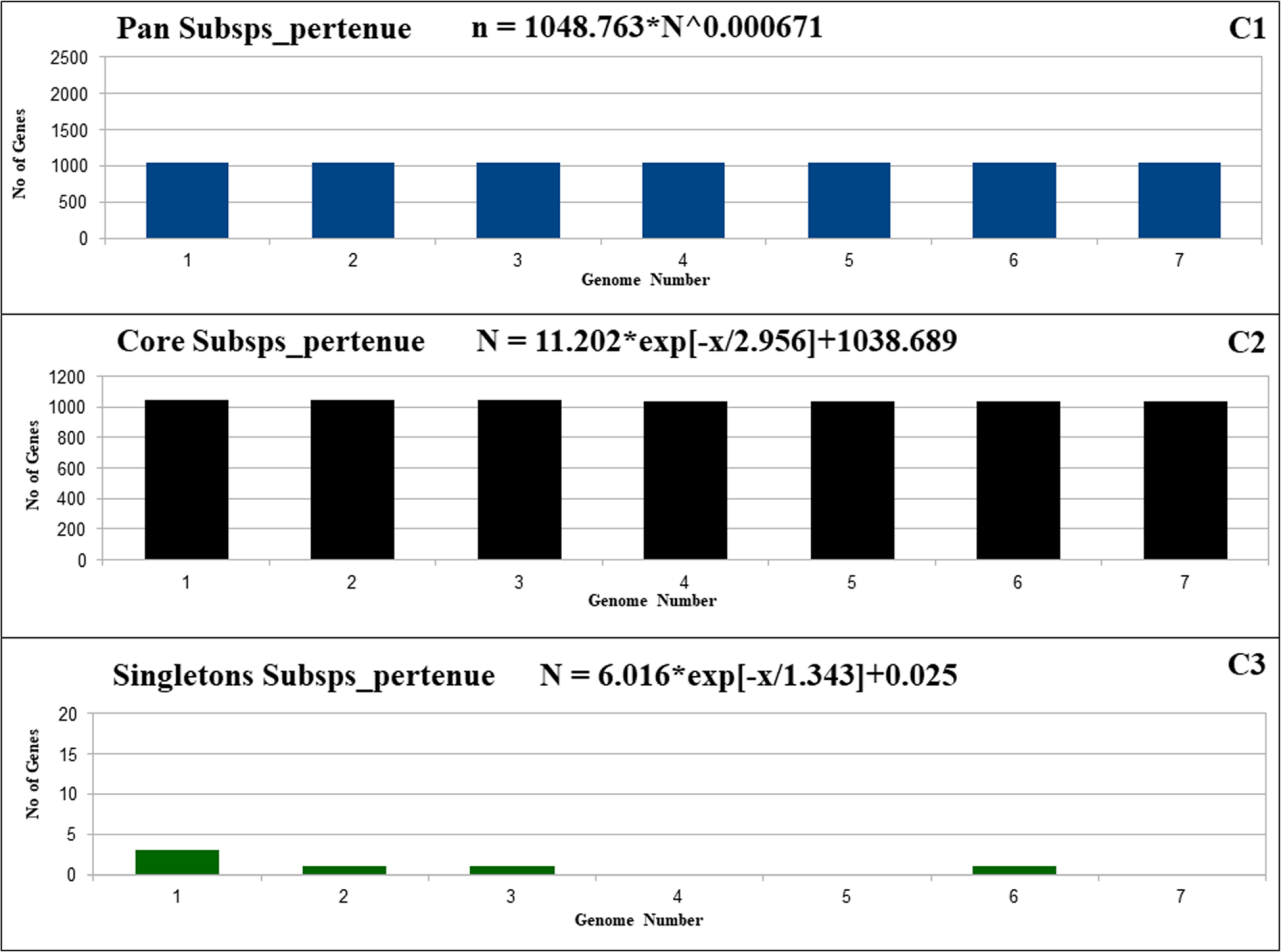
Pan-genome, core genome and singletons of *T. pallidum* Pan_subsp_pertenue. C1/C2/C3, respectively, showing the pan-genome, core genome and singletons development using 7 strains belonging to subspecies *pertenue*

The main goal of the pan-genome is the comparison of different strains of the same species or even genus at the genomic level. The resulting pan-genome of Pan All (Fig. 2A1-A3), Pan Subsp_pallidum (Fig. 3B1-B3), and Pan_subsp_pertenue (Fig. 4C1-C3), of *T. pallidum* contains a total of 2112, 982, and 1049 genes respectively. The formula (α =1-γ) inferred that the pan-genome of *T. pallidum* is increasing with an α of 0.9435. The extrapolation was also separately calculated for all divided subsets for the analysis in this work. The α value for each subset Pan Subsp_pallidum and Pan_subsp_pertenue, were 0.916 and 0.999329 respectively. The α values for all datasets used in this work are less than 1 which indicates that all have an open pan-genome. However, although the pan-genome is still open, it increases at a very low rate [20, 21].

The core genome and singletons of the complete dataset and all the subsets of *T. pallidum* were calculated by the least-squares fit of the exponential regression decay to the mean values, as represented by the formula n = k * exp[―x/τ*] + tg(θ)*, where *n* is the expected subset of genes for a given number of genomes, *x* is the number of genomes, *exp* is Euler’s number, and the other terms are constants defined to fit the specific curve. The resulting core genome of the complete dataset (Pan All), the subsets Pan Subsp_pallidum and Pan Subsp_pertenue, have the following *tg(θ)* values, respectively: *~* 318, *~* 627, and *~* 1038. Concerning the Singletons of the complete dataset (Pan All) and the subsets Pan Subsp_pallidum, and Pan Subsp_pertenue, have the following *tg(θ)* values, respectively: *~* 1, *~* 0.1, and *~* 0.025. *According to the least-squares fit of the exponential regression decay, the tg(θ) represents the point where the curve stabilizes, which may be translated to the number of genes in the core genome after stabilization and the number of singletons that will be added to the pan-genome for each newly sequenced genome. Considering this rule, the core genome of the subset* Subsp_pertenue *have higher number of core genes (1038-number of core genes) after stabilization, whereas, the complete dataset haS the smallest number of core genes (318-number of core genes). For the Singletons,* the *tg(θ*) *value for all the dataset indicates only one gene* will be added*, whereas, the subsets from* Pan Subsp_pallidum and Pan Subsp_pertenue will have 1 and 0.025 newly added genes respectively.

**Fig. 5:**
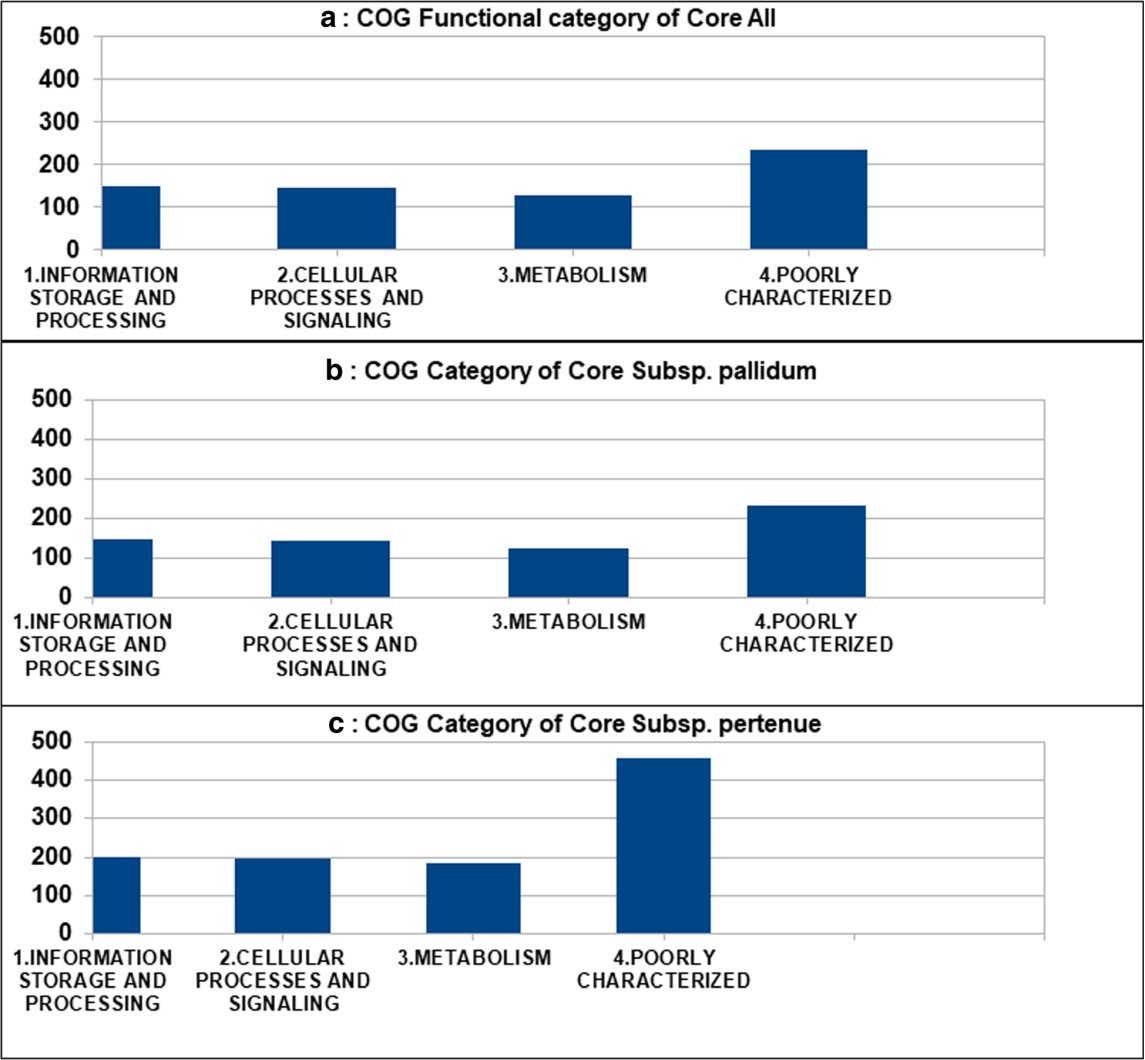
Graphical representation of COG (Cluster of Orthologous Genes) functional categories of identified core genes. **a**, **b**, **c** showing the core genes belonging to the Information storage and processing, cellular processing and signaling, Metabolism and poorly characterized functional categories for complete dataset, the subsets Pan Subsp_pallidum and Pan Subsp_pertenue of *T. pallidum,* respectively

The core genes of the complete dataset, the subsets Pan Subsp_pallidum and Pan Subsp_pertenue, of *T. pallidum* were classified by COG (Cluster of Orthologous Genes) functional category. According to the chart in Fig. 5a-c, the core genome of all the strains had many genes related to the “Metabolism” and “Information storage and processing” categories. Moreover, the majority of the core genome of all the strains were classified as “poorly characterized” (Additional file 1: Table S2A-C).

### Detection of PAIs in the *Treponema pallidum* genome

**Fig. 6:**
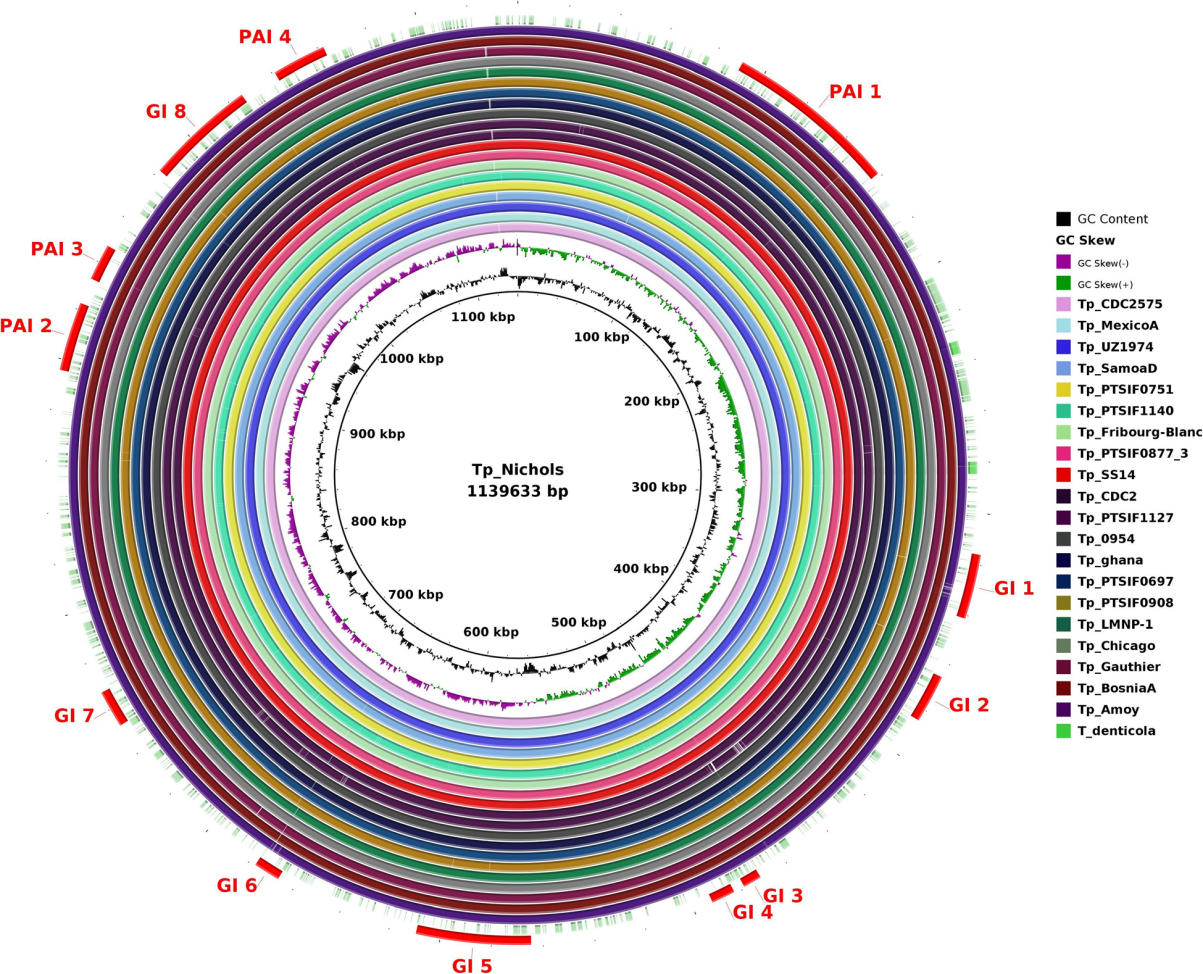
Circular genomic representation of islands (PAIs and GIs) in the genomes of *T. pallidum* subsp. *pallidum* strain Nichols as a reference. All genomes were aligned using strain Tp_Nichols as a reference. From the inner to outer circle the strains were represented in different colours. The outer most dotted circle represents the genome of *T. denticola*. The figure represents the coding sequences (CDS); GIs (Genomics islands) and PAIs (Pathogenicity Island)

**Fig. 7:**
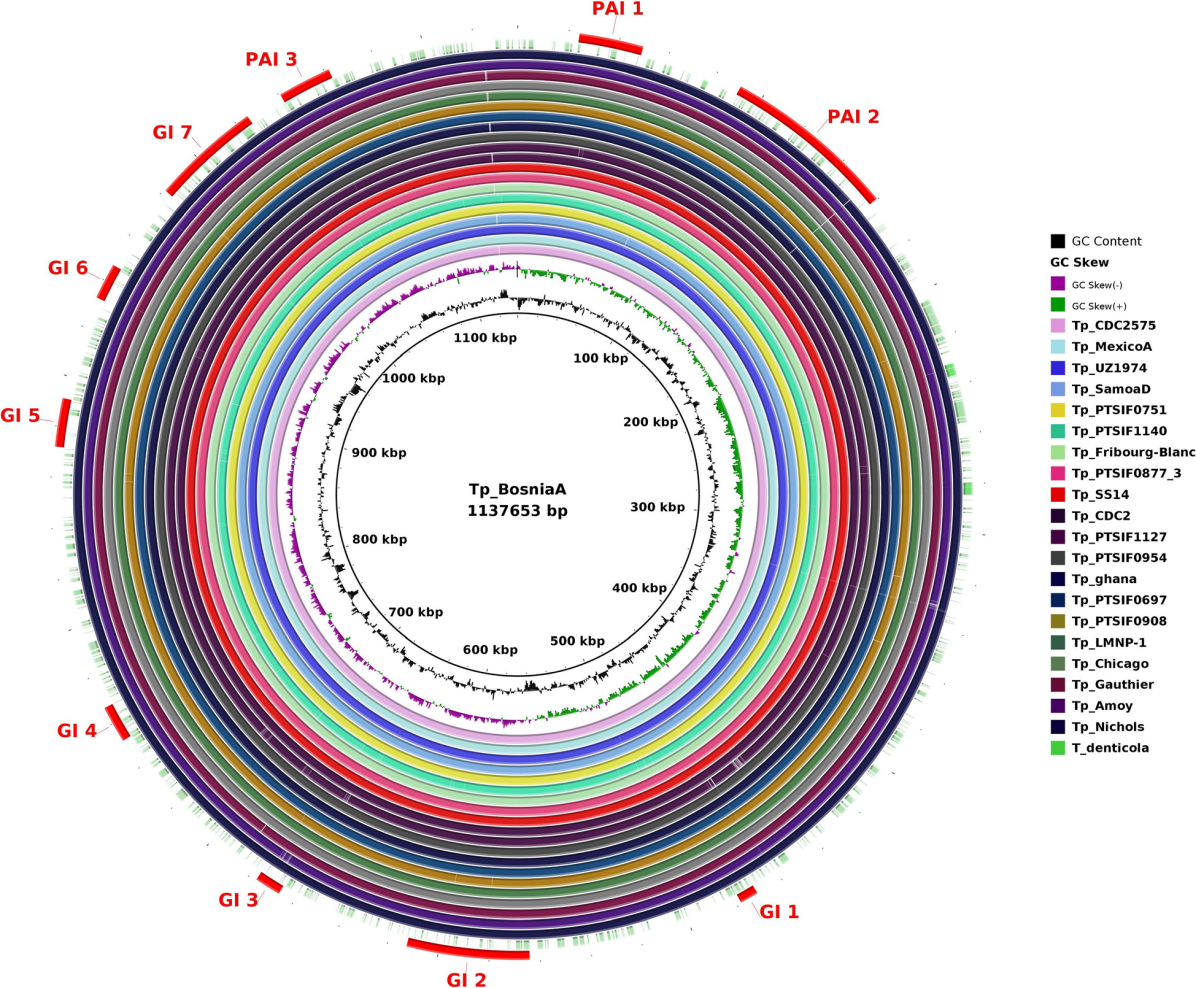
Circular genomic representation of islands (PAIs and GIs) in the genomes of *T. pallidum* subsp. *pertenue* strain SamoaD as a reference. All genomes were aligned using strain Tp_SamoaD as a reference. From the inner to outer circle the strains were represented in different colours. The outer most dotted circle represents the genome of *T. denticola*. The figure represents the coding sequences (CDS); GIs (Genomics islands) and PAIs (Pathogenicity Island)

**Fig. 8:**
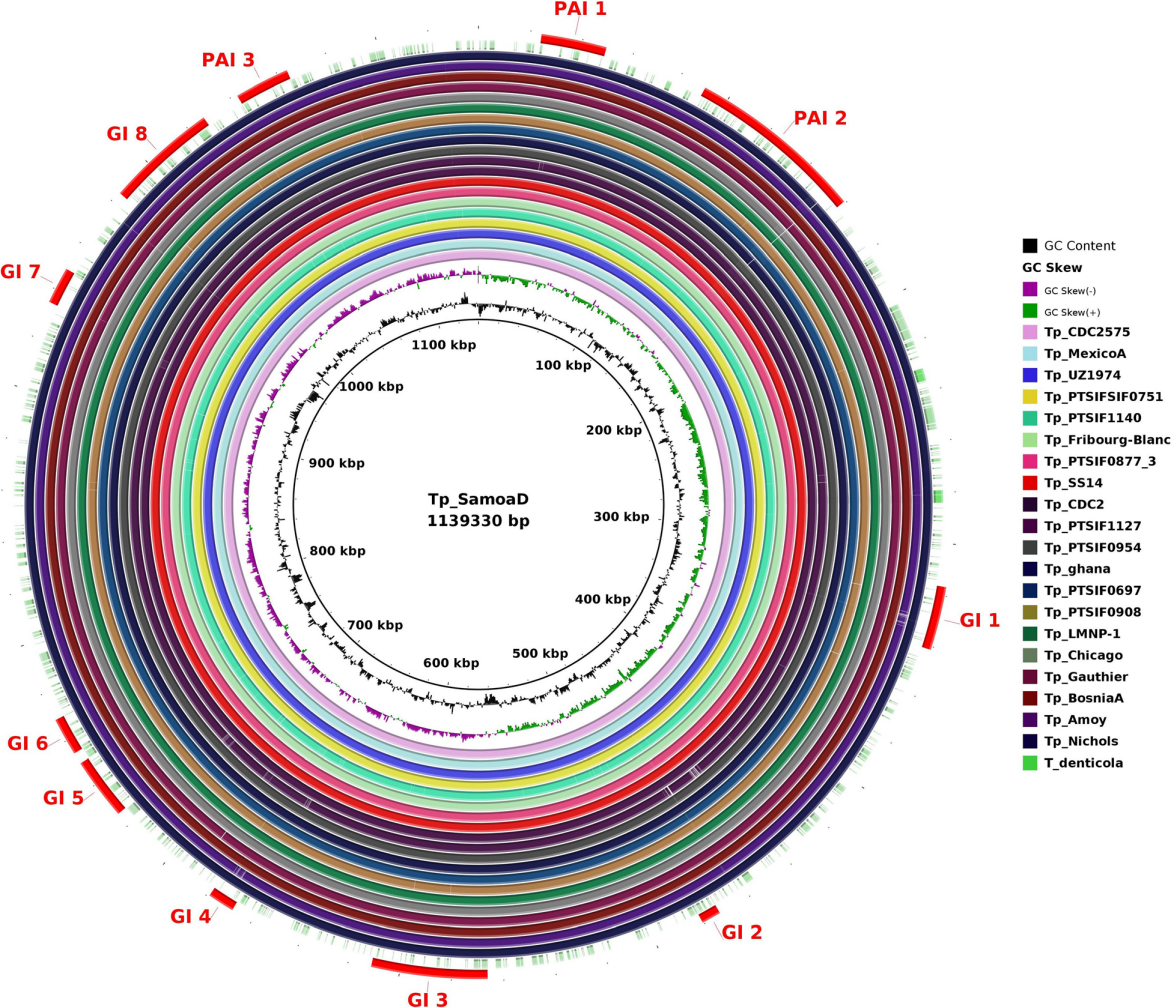
Circular genomic representation of islands (PAIs and GIs) in the genomes of *T. pallidum* subsp. *endemicum* strain BosniaA as a reference. All genomes were aligned using strain Tp_BosniaA as a reference. From the inner to outer circle the strains were represented in different colours. The outer most dotted circle represents the genome of *T. denticola*. The figure represents the coding sequences (CDS); GIs (Genomics islands) and PAIs (Pathogenicity Island)

The presence of pathogenicity islands (PAIs) is generally related to evolution in a different genomic environment [22]. However, it may only be the effect of relaxation of purifying selection genes involved in increasing the range of environmental responses. Interspecies genome plasticity may result from several events, of which horizontal gene transfer is particularly important because it can cause the acquisition of blocks of genes (genomic islands, or GIs), producing evolution by quantum leaps [23]. These genes are often flanked by transposases (insertion elements), have altered G + C content and skew, suggesting their acquisition through Horizontal Gene Transfer (HGT), intermediated by phages or recombination [22]. PAIs are important in this context because they represent a class of GIs that carry virulence genes, i.e., factors that enable or enhance the parasitic growth of an organism inside a host [24]. The genome plasticity of all 53 *T. pallidum* strains was determined by using GIPSy (Genomic Island Prediction Software) on subspecies-based study. The software BRIG (BLAST Ring Image Generator) [25] was used for the circular genome comparison visualization. Some of the other strains from the representing cluster of the dendogram were also used for the circular genome visualization. We found differences in the presence/absence of pathogenicity islands (PAIs) and genomic islands (GIs) on subspecies-based study: four Pathogenicity Islands (PAIs) eight genomic islands (GIs) in subsp. *pallidum* (Fig. 6); three PAIs and seven GIs in subsp. endemicum (Fig. 7); and, three PAIs and eight GIs in subsp. *pertenue* (Fig. 8).

### Variations in pathogenicity and Genomic Island in subspecies group

Regarding the presence of genes in PAIs and GIs, we compared the genes in all the subsp. of *T. pallidum* to each other. When compared to each other, we found high similarity of the genes in all the subsp. of *T. pallidum*. The genomic region related to PAIs 2 and PAIs 3 of subsp. *pertenue* and *endemicum* (Non- venereal subsp.) were similar to the PAIs 1 and PAIs 4 of subsp. *Pallidum*. When we compared the genes related to PAIs 2 of subsp. *pertenue* and *endemicum*, there were differences of three genes found that were only present in subsp. *pertenue*. Out of those three genes, two were hypothetical proteins and one was RNA polymerase sigma factor. Furthermore, the genes clusters related to the PAIs 3 of subs. *Pertenue* and *endemicum* were similar to PAIs 4 of subsp. *Pallidum.* Interestingly, we found the genomic region related to PAIs 1 of subsp. *pertenue* and *endemicum* (Non- venereal subsp.) were not present in any of the GIs or PAIs of subsp. *pallidum*. The list of genes related to PAI 1 of subsp. *pertenue* and *endemicum* is mentioned in Table 1**.**

**Table 1:** The list of genes related to PAI 1 of subs. *Pertenue* and *endemicum*

Protein ID	PAIs Coordinates	Protein Name	Functions (MF: Molecular Function, BP: Biological Process)
WP_012460510.1	22,054–24,177	VWFA domain-ontaining protein	–
WP_010881470.1	25,115–2538	Hypothetical protein	
WP_014342234.1	25,459–27,504	Hypothetical protein	–
WP_010881472.1	27,565–28,896	Sodium-dependent tryptophan transporter	MF: Neurotransmitter:sodium symporter activity BP
WP_010881473.1	29,076–29,786	Potassium transporter Trk	MF: Cation transmembrane transporter activity BP: Potassium ion transport
WP_010881474.1	29,865–32,936	M16C subfamily peptidase	MF: Catalytic activity, Metal ion binding BP: Proteolysis
WP_010881475.1	32,965–33,987	Flagellar motor switch protein FliG	MF: Motor activity BP: Bacterial-type flagellum-dependent cell motility, Chemotaxis
WP_014342235.1	34,060–35,433	Putative hemolysin HlyC	MF: Flavin adenine dinucleotide binding BP
WP_010881477.1	35,447–36,808	Putative hemolysin HlyC	MF: Flavin adenine dinucleotide binding BP
WP_039487502.1	36,916–38,193	UDP-N-acetylglucosamine 1-carboxyvinyltransferase	–
WP_010881479.1	38,312–39,946	60 kDa chaperonin	MF: ATP binding Source, Unfolded protein binding BP: Protein refolding
WP_014342237.1	40,010–40,288	Hypothetical protein	
WP_010881481.1	40,285–41,085	Ribosomal RNA small subunit methyltransferase E	MF: Methyltransferase activity BP: rRNA processing
WP_014342238.1	41,082–41,750	Hypothetical protein	
WP_039486943.1	41,794–42,924	Zinc (Zn2+) ABC superfamily ATP binding cassettetransporter, binding protein	MF: Metal ion binding BP: Cell adhesion, Metal ion transport
WP_010881484.1	42,942–43,658	Zinc (Zn2+) ABC superfamily ATP binding cassettetransporter, ABC protein	MF: ATPase activity, ATP binding BP
WP_010881485.1	43,658–44,458	Zinc (Zn2+) ABC superfamily ATP binding cassettetransporter, membrane protein	MF: ATPase-coupled transmembrane transporter activity BP:
WP_010881486.1	44,567–45,562	Lactate dehydrogenase	MF: D-lactate dehydrogenase activity, NAD binding BP
WP_014342240.1	45,659–46,711	Putative regulatory protein PfoR	MF: Protein-N (PI)-phosphohistidine-sugar phosphotransferase activity BP: Phosphoenolpyruvate-dependent sugar phosphotransferase system
WP_014342241.1	46,739–46,918	Hypothetical protein	
WP_039486948.1	46,945–49,392	Putative methyl-accepting chemotaxis protein	MF: Transmembrane signaling receptor activity BP: Chemotaxis, Signal transduction
WP_010881491.1	49,513–50,445	Peptidoglycan-binding protein LysM	–

On the other hand, we found that the genes present in PAIs 2 of subsp. *pallidum* were not present in any of the GIs or PAIs of subsp. *pertenue* and *endemicum* (Non- venereal subsp.). This may reflect the fact that the genomic signature of those regions has already adapted in subsp. *pallidum* to cause different modes of transmission. The list of genes related to PAI 2 of subsp. *pallidum* is mentioned in Table 2 excluding the hypothetical genes.

**Table 2:** The list of genes related to PAI 2 of subsp. *pallidum*

Protein ID	PAIs Coordinates	Protein Name	Functions (MF: Molecular Function, BP: Biological Process)
WP_010882272.1	895,372–895,749	holo-ACP synthase	MF: holo-[acyl-carrier-protein] synthase activity, magnesium ion binding. BP: Fatty acid biosynthetic process
WP_010882273.1	895,746–896,630	membrane protein	
WP_010882275.1	897,479–899,248	arginine--tRNA ligase	MF: arginine-tRNA ligase activity, ATP binding BP: arginyl-tRNA aminoacylation
WP_010882284.1	910,864–913,113	MFS transporter	
WP_010882286.1	914,944–915,711	methionine aminopeptidase	Aminopeptidase activity, metal ion binding, metalloexopeptidase activity
WP_010882287.1	915,835–916,659	Heat shock protein, putative	
WP_010882288.1	916,752–917,804	glyceraldehyde-3-phosphate dehydrogenase	MF: glyceraldehyde-3-phosphate dehydrogenase (NAD+) phosphorylating activity. BP: glucose metabolic process, glycolytic process.
WP_010882292.1	919,085–919,453	50S ribosomal protein L20	MF: rRNA binding, structural constituent of ribosome. BP: translation.
WP_010882293.1	919,486–919,686	50S ribosomal protein L35	MF: structural constituent of ribosome, BP: translation.
WP_010882295.1	920,441–922,615	DUF4954 domain-containing protein	
WP_010882296.1	922,644–923,642	prolipoprotein diacylglyceryl transferase	MF: transferase activity, transferring glycosyl groups. BP: lipoprotein biosynthetic process.

Moreover, we also compared GIs of all subspecies; as a result, we found that the genes of some GIs which are present in the GI2 and GI4 in *pallidum* subspecies and are not reported in any of GIs of the subspecies *endemicum* and *pertenue* (Table 3). Most of the genes present in GI2 and GI4 of *pallidum* subspecies are hypothetical genes but some genes are chemotaxis protein (CheA) that are associated with the transmission of sensory signals from the chemoreceptors to the flagellar motors [26]. The mechanisms by which *T. pallidum* sense and respond to nutrient gradients help in pathogenic processes such as crossing the endothelial barrier to reach the bloodstream.

**Table 3:** The list of genes related to GI 2 and GI 4 of subsp. *pallidum.* The table shows the list of proteins excluding the hypothetical proteins

Protein ID	GI 2 and GI 4 Coordinates	Protein Name	Functions (MF: Molecular Function, BP: Biological Process)
WP_010881790.1	365,067–365,627	cytidylate kinase	MF: ATP binding, cytidylate kinase activity BP: pyrimidine nucleotide metabolic process.
WP_010881791.1	365,621–366,454	adenine glycosylase	MF: catalytic activity. DNA binding. BP: base-excision repair.
WP_010881792.1	366,459–369,881	transcription-repair coupling factor	MF: ATP binding, damaged DNA binding, hydrolase activity. BP: regulation of transcription, DNA-template, transcription-coupled nucleotide-excision repair.
WP_010881793.1	370,034–371,125	phospho-N-acetylmuramoyl-pentapeptide- transferase	MF: phospho-N-acetylmuramoyl-pentapeptide-transferase activity, UDP-N-acetylmuramoyl-L-alanyl-D-glutamyl-meso-2, transferase activity. BP:cell cycle, cell divisio, cell wall organization.
WP_010881797.1	373,895–374,425	FKBP-type peptidyl-prolyl cis-trans isomerase	MF: Metal ion binding, peptidyl-prolyl sis-trans isomerase activity. BP: protein refolding
WP_010881798.1	374,639–375,925	gamma-glutamyl-phosphate reductase	MF: glutamate-5semialdehyde dehydrogenase activity. BP: L-proline biosynthetic process.
WP_010881799.1	375,922–376,812	glutamate 5-kinase	MF: ATP-binding BP: L-proline biosynthetic process.
WP_010881801.1	377,198–377,707	ribonuclease H	MF: Metal-ion binding, nucleic acid binding
WP_010881802.1	377,970–378,596	thymidylate kinase	MF: ATP binding, thymidylate kinase activity. BP: dTDP biosynthetic process
WP_010881806.1	377,970–378,596	glycosyl hydrolase	MF: catalytic activity. BP: carbohydrate metabolic process.
WP_014342391.1	382,926–383,729	lysophosphatidic acid acyltransferase	MF: transferase activity. BP: metabolic process
WP_014505476.1	384,584–387,016	chemotaxis protein CheA	MF: ATP bindinf, phosphorelay sensor kinase activity BP: chemotaxis.
WP_010881907.1	488,333–488,911	SMC-Scp complex subunit ScpB	BP: cell division, chromosome separation
WP_014342436.1	488,928–489,725	RsuA family pseudouridine synthase	MF: pseudouridine synthase activity, RNA binding.
WP_010881910.1	490,476–490,835	transcriptional regulator	
WP_010881913.1	492,278–493,030	tRNA (guanine-N(7)-)-methyltransferase	MF: tRNA (guanine-N7-)-methyltransferase activity.

## Discussion

The subspecies *T. pallidum* subsp. *endemicum* (TEN) and *T. pallidum* subsp. *pertenue* (TPE), are reasons for the diseases bejel and yaws, respectively. In the last few years, *T. pallidum* subsp. *pallidum* (TPA), has been reported as a reemerging pathogen [1, 15]. These three subsp. of *Treponema pallidum* are so close to each other that they cannot be differentiated serologically, their morphology is indistinguishable and are antigenically cross-reactive [27, 28]. Mostly, the disease phenotype caused by these pathogens can only be distinguished clinically and geographically. The distribution of venereal syphilis is global, non-venereal yaws usually effect kids in hot and/or humid regions of Africa and Asia, endemic syphilis be in dry places like Sahelian Africa and Saudi Arabia [27, 29]. The nature of *T. pallidum* is highly invasive. It circulates through bloodstream and lymphatics and overruns a wide-ranging of tissues and organs. As demonstrated by the widespread clinical manifestations related to syphilis infections, *Treponema pallidum* subsp. *pallidum* crosses placental, endothelial and blood-brain barriers early in infection, the incidence of congenital syphilis and invasion of central nervous system has been observed in almost 40% of early syphilis patients. Though, the understanding of the mechanisms responsible for the widespread distribution capability of *T. pallidum* is still very limited [30, 31].

The transmission of yaws is characterized by direct contact on skin and primary cutaneous lesion. It is facilitated by damaged skin surface. Scratching or rubbing these damaged parts of the body can facilitate the lesions spread across the body [28, 29]. Contrarily, endemic syphilis is an acute infection. Primary lesions of endemic syphilis can be seen in the children of ages between 2 and 15 years in dry and arid climates. While the mode of transmission is not known, it is believed that it may occur through mucosal and skin contact, even via shared eating utensils or drinking vessels [28, 29].

The defined relationships among the bacteria are still argued. The expansion of next-generation sequencing (NGS) in last few decades influences the fields of treatment and prevention, especially about bacterial diseases [32]. The ability of genomics data of *T. pallidum* gives us better understanding of the biology involving its interaction with its hosts. A comprehensive in silico pan-genome study was carried out for 53 sequenced genomes of *T. pallidum*, which indicates that the pan-genome of *T. pallidum* is still open; however, it is increasing at a very low rate as represented by the α of 0.9435 for the Pan All and the α of 0.916 and 0.999329 for Pan Subsp_pallidum and Pan_subsp_pertenue, respectively. Moreover, the α of 0.999329 indicates that the Pan Subsp pertenue is almost closed, which is corroborated by the *tg(θ) of ~ 0.025.*

The genome plasticity analysis reveals the differences in the presence and absence of some genome regions when compared at the subspecies level. Pathogenicity islands carry the genes related to the virulence, which are essential and characterize a class of Genomics Island [33]. The comparative analysis of PAIs and GIs showed the absence of genes at the subspecies level. We found gene clusters, that are related to amino acid and lipid biosynthesis, belonging to PAIs 2 of *T. pallidum* subsp. *pallidum* have not been identified in any PAIs or GIs of *T. pallidum* subsp. *endemicum* and *T. pallidum* subsp. *pertenue*. It might be possible that these genes help bacteria to execute different modes of infection at subsp. level of *T. pallidum*. Acyl carrier protein (ACP) synthase (AcpS) catalyzes the transfer of the 4′-phosphopantetheine moiety from coenzyme A (CoA) onto a serine residue of apo-ACP, to convert apo-ACP to the functional holo-ACP. During the biosynthesis of fatty acids and phospholipids, the holo form of bacterial ACP plays a vital role in mediating the transfer of acyl fatty acid. AcpS is therefore an attractive target for therapeutic interpolation. It has been reported that, AcpS enzymes from *Mycoplasma pneumoniae* and *S. pneumoniae* may play a crucial role in the acylation of fatty acids derived from human tissues for their lipid biosynthesis, suggesting that AcpS is a more striking antimicrobial target for discovery of novel antibiotics than bacterial fatty acid biosynthetic enzymes [34, 35].

Moreover, the presence of chemotaxis protein (CheA) in different GIs of *T. pallidum* subsp. *pallidum* might be responsible for different molecular modes of infection as *T. pallidum* genome contains two operons for the Che response regulators [31, 36]. The bacterial transcription-repair coupling factor (TRCF) is a large, multi-domain, SF2 ATPase that is generally conserved. It forms the dual of nucleotide excision repair with transcription by dislodging inactive RNA polymerase molecules stalled at template DNA lesions, and by increasing the rate at which the Uvr(A) BC exonuclease acts at these sites [37].

Pathogens are frequently using antigenic variation mechanisms to elude the adaptive immune response that ultimately results in persistent infection [38]. It might be because of the variation in expression of different Tpr proteins in the syphilis spirochete, *Treponema pallidum* subsp. *pallidum*, that have important implications in its ability to elude host immune detection [39]. A 12-membered protein family *Treponema pallidum* repeat (tpr) has been identified in *T. pallidum* subsp. *Pallidum*, which may be concerned in the pathogenesis of *T. pallidum* [38, 40]. On the basis of amino acid homology, these 12 Tprs are further divided into three subfamilies: subfamily I (TprC, D, F, I), subfamily II (TprE, G, J), and subfamily III (TprA, B, H, K, L) [40] [41].

Despite the host’s efforts to eliminate the infection, mechanisms of *T. pallidum’s* persistence include residence within intracellular or immune-privileged positions to hide from the immune effectors. *T. pallidum‘s* has the ability to cape its surface with host serum proteins or mucopolysaccharides to dodge immune response and immunosuppression of the host triggered by syphilis infection [38]. Freeze-fracture electron microscopy of *T. pallidum* has revealed lack of integral membrane proteins in the outer membrane (OM) of *T. pallidum,* conceivably accounting for the reasonably poor antigenicity of this spirochete’s surface [38, 42, 43].

However, as *T. pallidum* could be phagocytized in the presence of opsonic antibody, antibody targets must be present on the surface of the bacterium. Furthermore, the treponemes harvested from the tissues of later stage infections after the elimination of majority of treponemes are resistant to opsonophagocytosis. It raised the likelihood of antigenic variation occurring in *T. pallidum*, but no exact variable antigen was identified [38, 44]. Following the identification and investigation of TprK, provides the first candidate antigen of *T. pallidum* that might function in fudging the immune response. TprK vary among and within *T. pallidum* strains, with diversity of sequence localized in seven distinct regions (V1-V7) bordered by conserved domains [38, 43, 45, 46]. During experimental infection, these V regions are the main targets of the host humoral immune response [38]. Antigenic variation of the TprK antigen has been acknowledged to explain the persistence of *T. pallidum* in the host.

Recent work of Dan Liu et al. [47] has recognized an improved number of variants within these seven V regions of the tprK gene in the samples of secondary syphilis. A 3-bp changing pattern was observed in the sequences within each V region of the protein. However, same pattern of change was observed in variable sequences within the V regions of tprK in the secondary syphilis. Notably, the amino acid sequences IASDGGAIKH and IASEDGSAGNLKH in V1 are not only present in high proportion in inter-strain comparison but also were found at a quite high frequency in the populations. The alignment of all amino acid sequences revealed some really stable pattern within each V region of the primary and secondary syphilis samples, particularly the amino acid sequences IASDGGAIKH and IASEDGSAGNLKH in V1 region. The highly stable peptides found in V1 region are likely promising vaccine components. The highly heterogenetic regions (e.g., V6) could help to understand the role of tprK in fudging immune response. However, in our analysis, we found that some of tpr genes (*tprC, tprD,tprF,tprI, trpJ*) were present in some of PAIs or GIs *T. pallidum* subsp. *endemicum* (TEN) and *T. pallidum* subsp. *pertenue* (TPE). While, the GIs and PAIs related to *T. pallidum* subsp. *pallidum* we only identified some tpr domain proteins. It has been reported by Maděrankova et al. 2019, tpr genes responsible for the adaptive evolution of the pathogen [48].

## Conclusions

Apart from establishing phylogenetic relationships among treponemal species and subspecies, the addition of comparative genomics was also required to illuminate the lower degree of virulence associated with *T. pallidum* subsp. *pertenue* than with *T. pallidum* subsp. *pallidum*. Unlike syphilis, it is said that yaws cannot be transmitted vertically or affect the central nervous system. It is rather limited to skin, bones, joints and soft tissues. In the 1980s, a very limited genetic diversity between these pathogens was established when hybridization experiments were carried out with DNA isolated from yaws and syphilis strains [29]. Our work also showed that genomes of syphilis, yaws, and Bejel treponemes share 97–100% overall similarity, as well as the identical organization. This evidence proposes that small genetic changes in key genes among these organisms could be responsible for the reported differences in disease pathogenesis. Considering the genes in PAIs and GIs, we identified some absence of pathogenicity islands in all subspecies. Genes which are present in *pallidum* subspecies pathogenicity islands (PAIs) or genomic islands (GIs) are absent in the subspecies *endemicum* and *pertenue*. The findings of this analysis are very important, as it can help in the understanding of molecular basis of infections from *T. pallidum* subsps. Furthermore, the core genes represent the most desirable source for the selection of conserved genes; therefore, characterization of such poorly studied proteins helps in understanding the cellular metabolism, mode of infection and regulation of gene expression of *Treponema pallidum*. Hence, this study can help to better understand the molecular modes of bacterial infection and are significance for vaccine development for syphilis.

## Methods

### Genome sequences

The genome sequences of 53 *T. pallidum* strains were retrieved from the NCBI (National Centre for Biotechnology Information) database (https://www.ncbi.nlm.nih.gov/genome/genomes/741?) (Accessed June 2018): 46 genomes of *T. pallidum* subsp. *pallidum* were isolated from different parts of human body, rabbits and baboons (USA, China & Portugal). Six genomes from Africa and Australia/ Oceania continents (strain SamoaD, CDC2, Gauthier, CDC2575, Ghana051 and LMNP-1) from subsp. *pertenue* were isolated from humans, baboons and rabbits (Additional file 1: Table S1). One genome of *Treponema pallidum* subsp. *endemicum* (strain BosniaA) was isolated in Europe from human tongue and tonsils. The genome of *Treponema denticola* strain ATCC 35405 was used as non-pathogenic bacteria in this work. The general information about all *T. pallidum* strains and the Complete workflow applied in this work are given in Additional file 1: Table S1 and Figure S1, respectively.

### Phylogenomic analysis of all *Treponema pallidum* strains

For phylogenomic analysis of all *Treponema pallidum* strains, Gegenees (version 2.1) [19] was used. The Gegenees software was used to perform an all-versus-all similarity search. It divides the genomes into small sequences and determines the minimum content shared by all the genomes. Subsequently, the obtained minimum shared contents were subtracted from all the genomes resulting in the variable contents, which were eventually compared with all the other strains for the calculation of the percentages of similarity. Finally, these percentages were plotted in a heatmap chart with a spectrum ranging from low similarity (red) to high similarity (green). The Gegenees data was exported as a distance matrix file in nexus format (.nex) and, further, the generated distance matrix was used as an input file in SplitsTree software (version 4.14.5) [49] using neighbour joining method to create a dendogram [50, 51].

### Prediction of Pan-genome, Core-genome and singleton

We divided 53 strains of *T. pallidum* in 3 subsets for pan-genome calculation. We performed Pan All (with all 53 strains of *T. pallidum*), Pan Subsp_pallidum and Pan_subsp_pertenue (based on subspecies). For the identification of core genome (commonly shared by all strains), shared genome (genes present in two or more than two strains but not shared by all strains) and singletons (strain specific genes), we used OrthoFinder [52]. Briefly, OrthoFinder uses the .faa amino acid sequence file for each genome to perform *all-*vs*-all* BLASTp for the Orthologous analysis. It uses MCL (Markov Clustering algorithm) program to determine the Orthologous genes [53]. The cut-off value of 1e^− 10^ was used for Pan-genome, Core-genome and singletons identification for all the subsets. Furthermore, *in-house* scripts were used to estimate the fixed parameters for Heap’s Law (pan-genome analyses) [20, 51] and least-squares fit of the exponential regression decay (core-genome and singletons). The extrapolations of the pan-genomes from the complete dataset and all subsets were calculated based on Heap’s Law [20, 51], which was used to calculate whether the pan-genome was open or closed. Heap’s Law is an empirical law represented by the formula n = k*N^γ^; it describes the number of distinct words in a document (or set of documents) as a function of the document length. In a genetic context, n is the expected number of genes for a given number of genomes, N determines the number of genomes, and the k and γ (α =1-γ) are free parameters that are determined empirically. According to Heap’s Law, when α > 1 (γ < 0), the pan-genome is considered to be closed, and there will be no significant increase in the number of genes with the addition of a new genome. On the other hand, when α < 1 (0 < γ < 1), the pan-genome is open and there will be a significant increase in the number of genes for each newly added genome.

### Genomic and Pathogenicity Islands prediction

This section describes the analyses that were performed for the prediction of genomic and pathogenicity Islands following three datasets based on the subspecies: A) using *T. pallidum* subsp. *pallidum* strain Nichols as a reference; B) using *T. pallidum* subsp. *pertenue* strain SamoaD as a reference; and C) using *T. pallidum* subsp. *endemicum* strain BosniaA as a reference. The islands predictions for three datasets were determined by using GIPSy (Genomic Island Prediction Software) [33]. GIPSy is a multi-step approach that predicts Genomic islands (GIs) and Pathogenicity islands (PAIs). PAIs and GIs predictions are based on commonly shared features such as genomic signature deviation (anomalous G + C content and codon usage deviation), presence of transposase genes; metabolism, virulence, antibiotic resistance, or symbiosis-related genes; flanking tRNA genes; and absence in other organisms of the same genus or closely related species [33]. *T. denticola* strain ATCC 35405 was used as a non-pathogenic species from the same *Treponema* genus for GIs and PAIs prediction [54]. The sizes of the islands were compared with all the other strains via ACT (Artemis Comparison Tool) software [55]. PAIs regions were plotted using the software BRIG [25]. Following the curation of the PAIs, the genes of all the islands in each strain were assessed for their presence/absence in all the other strains.

## Supplementary information

**Additional file 1: Table S1.** General information about 53 *Treponema pallidum* Strains used in this work. List of all *Treponema pallidum* strains (with features) retrieved from the NCBI (National Center for Biotechnology Information) database. **Table S2A.** The COG functional categories with detailed description of Core genes: The table showing the number of core genes of the complete dataset were classified by COG (Cluster of Orthologous Genes) functional category. **Table S2B.** The COG functional categories with detailed description of Core genes: The table showing the number of core genes of the Pan Subsp_pallidum dataset were classified by COG (Cluster of Orthologous Genes) functional category. **Table S2C.** The COG functional categories with detailed description of Core genes: The table showing the number of core genes of the Pan Subsp_pertenue dataset were classified by COG (Cluster of Orthologous Genes) functional category. **Figure S1.** The Complete workflow applied in this work. The figure represent the methodology and software were used in this analysis. **Figure S2.** The heatmap analysis of 53 Strains of *Treponema pallidum.*The figure represents the comparison between the variable content of all strains. The percentages were plotted in the heatmap with a spectrum ranging from red (low similarity) to green (high similarity). The names of the strains on the left side of the figure (vertically) are organized in the same order in the top part of the figure (horizontally). Once Gegenees uses the similarities in the variable contents, the outgroup normally presents a very small percentage of similarity to the other strains.

## Data Availability

All data generated and analysed during this study are included in this published article and its supplementary information files.

## Abbreviations

CDS: Coding sequences
COG: Cluster of Orthologous Genes
GIs: Genomic islands
PAIs: Pathogenicity islands
TEN: *Treponema pallidum* subsp. *endemicum*
TPA: *Treponema pallidum* subsp. *pallidum*
TPE: *Treponema pallidum* subsp. *pertenue*
WHO: World Health Organization
