# Supplementary material for: The pan-genome of Treponema pallidum reveals differences in genome plasticity between subspecies related to venereal and non-venereal syphilis
Source: BMC Genomics. 2020 Jan 10;21:33. doi: 10.1186/s12864-019-6430-6 (PMC6953169; doi:10.1186/s12864-019-6430-6)
Supplement: Supplementary file 1 — Additional file 1: Table S1. General information about 53 Treponema pallidum Strains used in this work. List of all Treponema pallidum strains (with features) retrieved from the NCBI (National Center for Biotechnology Information) database. Table S2A. The COG functional categories with detailed description of Core genes: The table showing the number of core genes of the complete dataset were classified by COG (Cluster of Orthologous Genes) functional category. Table S2B. The COG functional categories with detailed description of Core genes: The table showing the number of core genes of the Pan Subsp_pallidum dataset were classified by COG (Cluster of Orthologous Genes) functional category. Table S2C. The COG functional categories with detailed description of Core genes: The table showing the number of core genes of the Pan Subsp_pertenue dataset were classified by COG (Cluster of Orthologous Genes) functional category. Figure S1. The Complete workflow applied in this work. The figure represent the methodology and software were used in this analysis. Figure S2. The heatmap analysis of 53 Strains of Treponema pallidum.The figure represents the comparison between the variable content of all strains. The percentages were plotted in the heatmap with a spectrum ranging from red (low similarity) to green (high similarity). The names of the strains on the left side of the figure (vertically) are organized in the same order in the top part of the figure (horizontally). Once Gegenees uses the similarities in the variable contents, the outgroup normally presents a very small percentage of similarity to the other strains. [file 12864_2019_6430_MOESM1_ESM.doc]

Additional file 1

**Table S1: General information about 53 *Treponema pallidum* Strains used in this work.** List of all *Treponema pallidum* strains (with features) retrieved from the NCBI (National Center for Biotechnology Information) database.

| **Strain** | **Size(Mb)** | **Subspecies** | **Geographical Location** | **Harvested** | **GenBank Accession No** | **GC%** | **Gene** | **Protein** |
| --- | --- | --- | --- | --- | --- | --- | --- | --- |
| Tp_Nichols | 1.13 | pallidum | Washington DC | Human- Neurosyphilitic patient | AE000520.1 | 52.80 | 1044 | 970 |
| Tp_Sea81-4 | 1.13 | pallidum | USA: Seattle. WA | Human | CP003679.1 | 52.80 | 1032 | 931 |
| Tp_SS14 | 1.13 | pallidum | San DiegoCA: USA | Human- Skin | CP004011.1 | 52.80 | 1066 | 1002 |
| Tp_Chicago | 1.13 | pallidum | San DiegoCA: USA | Rabbit- testis | CP001752.1 | 52.80 | 1030 | 969 |
| Tp_SamoaD | 1.13 | pertenue | Australia/Oceania | Rabbit-testis | CP002374.1 | 52.80 | 1064 | 1005 |
| Tp_CDC2 | 1.13 | pertenue | Africa | Human | CP002375.1 | 52.80 | 1030 | 973 |
| Tp_Gautheir | 1.13 | pertenue | Africa | Human- Skin | CP002376.1 | 52.80 | 1029 | 971 |
| Tp_DAL1 | 1.13 | pallidum | Africa | Human- | CP003115.1 | 52.80 | 1030 | 969 |
| Tp_MexicoA | 1.14 | pallidum | Mexico | Human- Skin | CP003064.1 | 52.80 | 1029 | 968 |
| Tp_Fribourg-Blanc | 1.14 | pertenue | West Africa | Baboons- Skin | CP003902.1 | 52.80 | 1030 | 970 |
| Tp_Nichols(2013/06/11) | 1.13 | pallidum | Washington DC | Human- Skin | CP004010.2 | 52.80 | 1065 | 1004 |
| Tp_SS14(11.12.2013) | 1.13 | pallidum | USA: Seattle. WA | Human- Skin | CP000805.1 | 52.80 | 1088 | 1028 |
| Tp_BosniaA | 1.13 | endemicum | Europe | Human-Tongue & tonsils. | CP007548.1 | 52.80 | 1065 | 1003 |
| Tp_pallidum Amoy | 1.13 | pallidum | China: Xiamen | Not Available | CP015162.1 | 52.70 | 1033 | 964 |
| Tp_Chicago population | 1.13 | pallidum | USA: Seattle | Human-Bacteria harveste from rabbit testes | CP010558.1 | 52.80 | 1034 | 971 |
| Tp_CDC-A | 1.13 | pallidum | USA: Seattle | Human-Bacteria harveste from rabbit testes | CP010559.1 | 52.80 | 1033 | 969 |
| Tp_Nichols Houston, Clone E | 1.13 | pallidum | USA: Seattle | Human-Bacteria harveste from rabbit testes | CP010560.1 | 52.80 | 1031 | 966 |
| Tp_Nichol Houston, Clone J | 1.13 | pallidum | USA: Seattle | Human-Bacteria harveste from rabbit testes | CP010561.1 | 52.80 | 1031 | 969 |
| Tp_UW074B | 1.13 | pallidum | USA: Seattle | Human-Blood | CP010562.1 | 52.80 | 1033 | 969 |
| Tp_UW189B | 1.13 | pallidum | USA: Seattle | Human-Blood | CP010563.1 | 52.80 | 1033 | 970 |
| Tp_UW228B | 1.13 | pallidum | USA: Seattle | Human-Blood | CP010564.1 | 52.80 | 1034 | 973 |
| Tp_UW254B | 1.13 | pallidum | USA: Seattle | Human-Blood | CP010565.1 | 52.80 | 1033 | 970 |
| Tp_UW391B | 1.13 | pallidum | USA: Seattle | Human-Blood | CP010566.1 | 52.80 | 1025 | 962 |
| Tp_PT_SIF0697 | 1.13 | pallidum | Portugal | Human- Penile | CP016045.1 | 52.80 | 1035 | 970 |
| Tp_PT_SIF0857 | 1.13 | pallidum | Portugal | Human-Oropharyngeal | CP016047.1 | 52.80 | 1035 | 973 |
| Tp_PT_SIF0908 | 1.13 | pallidum | Portugal | Human- Vaginal | CP016049.1 | 52.80 | 1034 | 933 |
| Tp_PT_SIF0954 | 1.13 | pallidum | Portugal | Human- Scrotum | CP016050.1 | 52.80 | 1034 | 970 |
| Tp_PT_SIF1002 | 1.13 | pallidum | Portugal | Human- Penile | CP016051.1 | 52.80 | 1033 | 970 |
| Tp_ PT_SIF1020 | 1.13 | pallidum | Portugal | Human- Penile | CP016052.1 | 52.80 | 1033 | 969 |
| Tp_ PT_SIF1063 | 1.13 | pallidum | Portugal | Human- Anal | CP016053.1 | 52.80 | 1035 | 972 |
| Tp_ PT_SIF1127 | 1.13 | pallidum | Portugal | Human- Penile | CP016054.1 | 52.80 | 1035 | 917 |
| Tp_ PT_SIF1135 | 1.13 | pallidum | Portugal | Human- Anal | CP016055.1 | 52.80 | 1036 | 972 |
| Tp_ PT_SIF1140 | 1.13 | pallidum | Portugal | Human- Anal | CP016056.1 | 52.80 | 1032 | 863 |
| Tp_ PT_SIF1142 | 1.13 | pallidum | Portugal | Human- Penile | CP016057.1 | 52.80 | 1035 | 971 |
| Tp_ PT_SIF1156 | 1.13 | pallidum | Portugal | Human- Penile | CP016058.1 | 52.80 | 1035 | 971 |
| Tp_ PT_SIF1167 | 1.13 | pallidum | Portugal | Human- Penile | CP016059.1 | 52.80 | 1035 | 971 |
| Tp_ PT_SIF1183 | 1.13 | pallidum | Portugal | Human- Penile | CP016060.1 | 52.80 | 1034 | 971 |
| Tp_ PT_SIF1196 | 1.13 | pallidum | Portugal | Human- Penile | CP016061.1 | 52.80 | 1037 | 974 |
| Tp_ PT_SIF1200 | 1.13 | pallidum | Portugal | Human- Penile | CP016062.1 | 52.80 | 1035 | 973 |
| Tp_ PT_SIF1242 | 1.13 | pallidum | Portugal | Human- Penile | CP016063.1 | 52.80 | 1034 | 971 |
| Tp_ PT_SIF1252 | 1.13 | pallidum | Portugal | Human- Penile | CP016064.1 | 52.80 | 1035 | 972 |
| Tp_ PT_SIF 1261 | 1.13 | pallidum | Portugal | Human- Anal | CP016065.1 | 52.80 | 1034 | 971 |
| Tp_ PT_SIF1278 | 1.13 | pallidum | Portugal | Human- Anal | CP016066.1 | 52.80 | 1034 | 972 |
| Tp_ PT_SIF1280 | 1.13 | pallidum | Portugal | Human- Anal | CP016067.1 | 52.80 | 1034 | 972 |
| Tp_ PT_SIF1299 | 1.13 | pallidum | Portugal | Human- Penile | CP016068.1 | 52.80 | 1035 | 972 |
| Tp_ PT_SIF1348 | 1.13 | pallidum | Portugal | Human- Penile | CP016069.1 | 52.80 | 1032 | 967 |
| Tp_ PT_SIF0751 | 1.13 | pallidum | Portugal | Human- Tongue | CP016046.1 | 52.80 | 1033 | 969 |
| Tp_ PT_SIF0877_3 | 1.13 | pallidum | Portugal | Human- Tongue | CP016048.1 | 52.80 | 1034 | 971 |
| Tp_seattle Nichols | 1.13 | Pallidum | USA Seattle WA | Human-Bacteria harveste from rabbit testes | CP010422.1 | 52.80 | 1065 | 1000 |
| Tp_Ghana-051 | 1.13 | pertenue | Ghana | Human | CP020365.1 | 52.80 | 1067 | 1006 |
| Tp_CDC 2575 | 1.13 | pertenue | Ghana | Human | CP020366.1 | 52.80 | 1067 | 1006 |
| Tp_UZ1974 | 1.13 | pallidum | Czeck Republic (Europe) | Human | CP028438.1 | 52.80 | 1066 | 1000 |
| Tp_LMNP-1 | 1.13 | pertenue | Tanzania | Papio anubis | CP021113.1 | 52.80 | 1064 | 1000 |

**Table S2A:** **The COG functional categories with detailed description of Core genes:** The table showing the number of core genes of the complete dataset were classified by COG (Cluster of Orthologous Genes) functional category.

| **Code** | **Description** | **No of Genes** | **Percentage** |
| --- | --- | --- | --- |
| **Information Storage and processing** | | | |
| [A] | RNA processing and modification | 0 | 0.0 |
| [B] | Chromatin structure and dynamics | 0 | 0.0 |
| [J] | Translation, ribosomal structure and biogenesis | 93 | 14.29 |
| [K] | Transcription | 19 | 2.91 |
| [L] | Replication, recombination and repair | 30 | 4.70 |
| **Cellular processes and signalling** | | | |
| [D] | Cell cycle control, cell division, chromosome partitioning | 11 | 1.69 |
| [M] | Cell wall/membrane biogenesis | 39 | 6.00 |
| [N] | Cell motility | 9 | 1.40 |
| [O] | Posttranslational modification, protein turnover, chaperones | 29 | 4.50 |
| [T] | Signal transduction mechanisms | 15 | 3.30 |
| [U] | Intracellular trafficking and secretion | 6 | 0.90 |
| [V] | Defense mechanisms | 5 | 0.80 |
| [W] | Extracellular structures | 0 | 0.0 |
| [Y] | Nuclear structure | 0 | 0.0 |
| [Z] | Cytoskeleton | 0 | 0.0 |
| **Metabolism** | | | |
| [C] | Energy production and conversion | 17 | 2.61 |
| [E] | Amino acid transport and metabolism | 11 | 1.70 |
| [F] | Nucleotide transport and metabolism | 10 | 1.53 |
| [G] | Carbohydrate transport and metabolism | 18 | 2.80 |
| [H] | Coenzyme transport and metabolism | 15 | 2.30 |
| [I] | Lipid transport and metabolism | 13 | 1.90 |
| [P] | Inorganic ion transport and metabolism | 21 | 3.22 |
| [Q] | Secondary metabolites biosynthesis, transport and catabolism | 0 | 0.0 |
| **Poorly Characterized** | | | |
| [R] | General function prediction only | 52 | 8.00 |
| [S] | Function unknown | 35 | 5.38 |
| - | Not in COGs | 203 | 31.18 |

**Table S2B: The COG functional categories with detailed description of Core genes:** The table showing the number of core genes of the Pan Subsp_pallidum dataset were classified by COG (Cluster of Orthologous Genes) functional category.

| **Code** | **Description** | **No of Genes** | **Percentage** |
| --- | --- | --- | --- |
| **Information Storage and processing** | | | |
| [A] | RNA processing and modification | 0 | 0.0 |
| [B] | Chromatin structure and dynamics | 0 | 0.0 |
| [J] | Translation, ribosomal structure and biogenesis | 95 | 14.54 |
| [K] | Transcription | 24 | 3.67 |
| [L] | Replication, recombination and repair | 33 | 5.05 |
| **Cellular processes and signalling** | | | |
| [D] | Cell cycle control, cell division, chromosome partitioning | 11 | 1.68 |
| [M] | Cell wall/membrane biogenesis | 43 | 6.58 |
| [N] | Cell motility | 27 | 4.13 |
| [O] | Posttranslational modification, protein turnover, chaperones | 36 | 5.51 |
| [T] | Signal transduction mechanisms | 28 | 4.28 |
| [U] | Intracellular trafficking and secretion | 18 | 2.75 |
| [V] | Defense mechanisms | 6 | 0.91 |
| [W] | Extracellular structures | 0 | 0.0 |
| [Y] | Nuclear structure | 0 | 0.0 |
| [Z] | Cytoskeleton | 0 | 0.0 |
| **Metabolism** | | | |
| [C] | Energy production and conversion | 25 | 3.82 |
| [E] | Amino acid transport and metabolism | 15 | 2.29 |
| [F] | Nucleotide transport and metabolism | 13 | 1.99 |
| [G] | Carbohydrate transport and metabolism | 25 | 3.82 |
| [H] | Coenzyme transport and metabolism | 18 | 2.75 |
| [I] | Lipid transport and metabolism | 15 | 2.29 |
| [P] | Inorganic ion transport and metabolism | 24 | 3.67 |
| [Q] | Secondary metabolites biosynthesis, transport and catabolism | 1 | 0.15 |
| **Poorly Characterized** | | | |
| [R] | General function prediction only | 61 | 9.34 |
| [S] | Function unknown | 36 | 5.51 |
| - | Not in COGs | 99 | 15.59 |

**Table S2C: The COG functional categories with detailed description of Core genes:** The table showing the number of core genes of the Pan Subsp_pertenue dataset were classified by COG (Cluster of Orthologous Genes) functional category.

| **Code** | **Description** | **No of Genes** | **Percentage** |
| --- | --- | --- | --- |
| **Information Storage and processing** | | | |
| [A] | RNA processing and modification | 0 | 0.0 |
| [B] | Chromatin structure and dynamics | 0 | 0.0 |
| [J] | Translation, ribosomal structure and biogenesis | 117 | 11.25 |
| [K] | Transcription | 22 | 2.11 |
| [L] | Replication, recombination and repair | 51 | 4.90 |
| **Cellular processes and signalling** | | | |
| [D] | Cell cycle control, cell division, chromosome partitioning | 13 | 1.25 |
| [M] | Cell wall/membrane biogenesis | 61 | 5.86 |
| [N] | Cell motility | 42 | 4.03 |
| [O] | Posttranslational modification, protein turnover, chaperones | 47 | 4.51 |
| [T] | Signal transduction mechanisms | 32 | 3.07 |
| [U] | Intracellular trafficking and secretion | 29 | 2.78 |
| [V] | Defense mechanisms | 7 | 0.67 |
| [W] | Extracellular structures | 0 | 0.0 |
| [Y] | Nuclear structure | 0 | 0.0 |
| [Z] | Cytoskeleton | 0 | 0.0 |
| **Metabolism** | | | |
| [C] | Energy production and conversion | 38 | 3.65 |
| [E] | Amino acid transport and metabolism | 27 | 2.59 |
| [F] | Nucleotide transport and metabolism | 21 | 2.01 |
| [G] | Carbohydrate transport and metabolism | 43 | 4.13 |
| [H] | Coenzyme transport and metabolism | 21 | 2.01 |
| [I] | Lipid transport and metabolism | 19 | 1.82 |
| [P] | Inorganic ion transport and metabolism | 26 | 2.5 |
| [Q] | Secondary metabolites biosynthesis, transport and catabolism | 3 | 0.28 |
| **Poorly Characterized** | | | |
| [R] | General function prediction only | 87 | 8.36 |
| [S] | Function unknown | 136 | 13.07 |
| - | Not in COGs | 198 | 19.03 |

**Figure S1.** **The Complete workflow applied in this work.** The figure represent the methodology and software were used in this analysis.

**
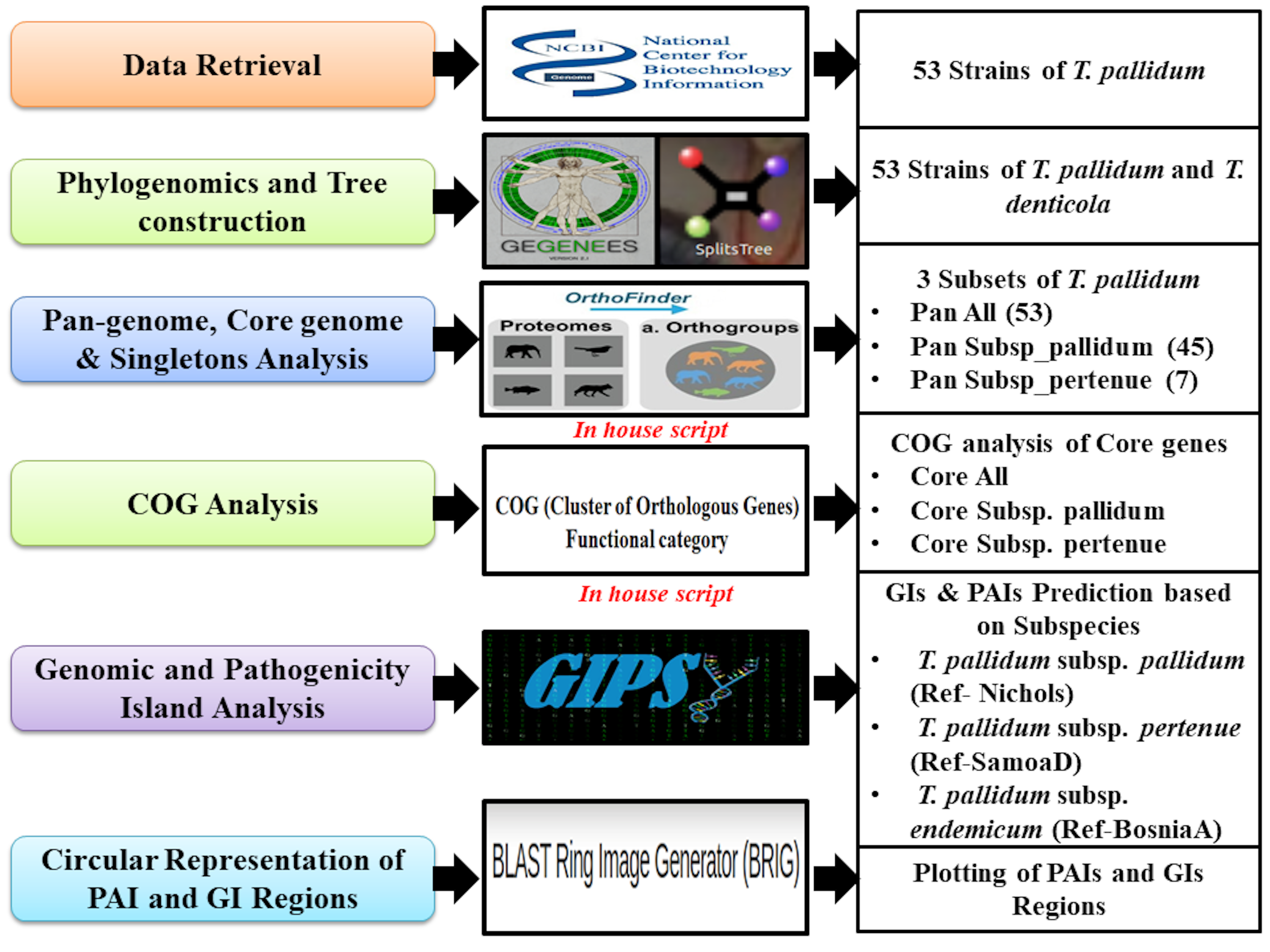
**

**Figure S2:** **The heatmap analysis of 53 Strains of *Treponema pallidum.***

The figure represents the comparison between the variable content of all strains. The percentages were plotted in the heatmap with a spectrum ranging from red (low similarity) to green (high similarity). The names of the strains on the left side of the figure (vertically) are organized in the same order in the top part of the figure (horizontally). Once Gegenees uses the similarities in the variable contents, the outgroup normally presents a very small percentage of similarity to the other strains.

**
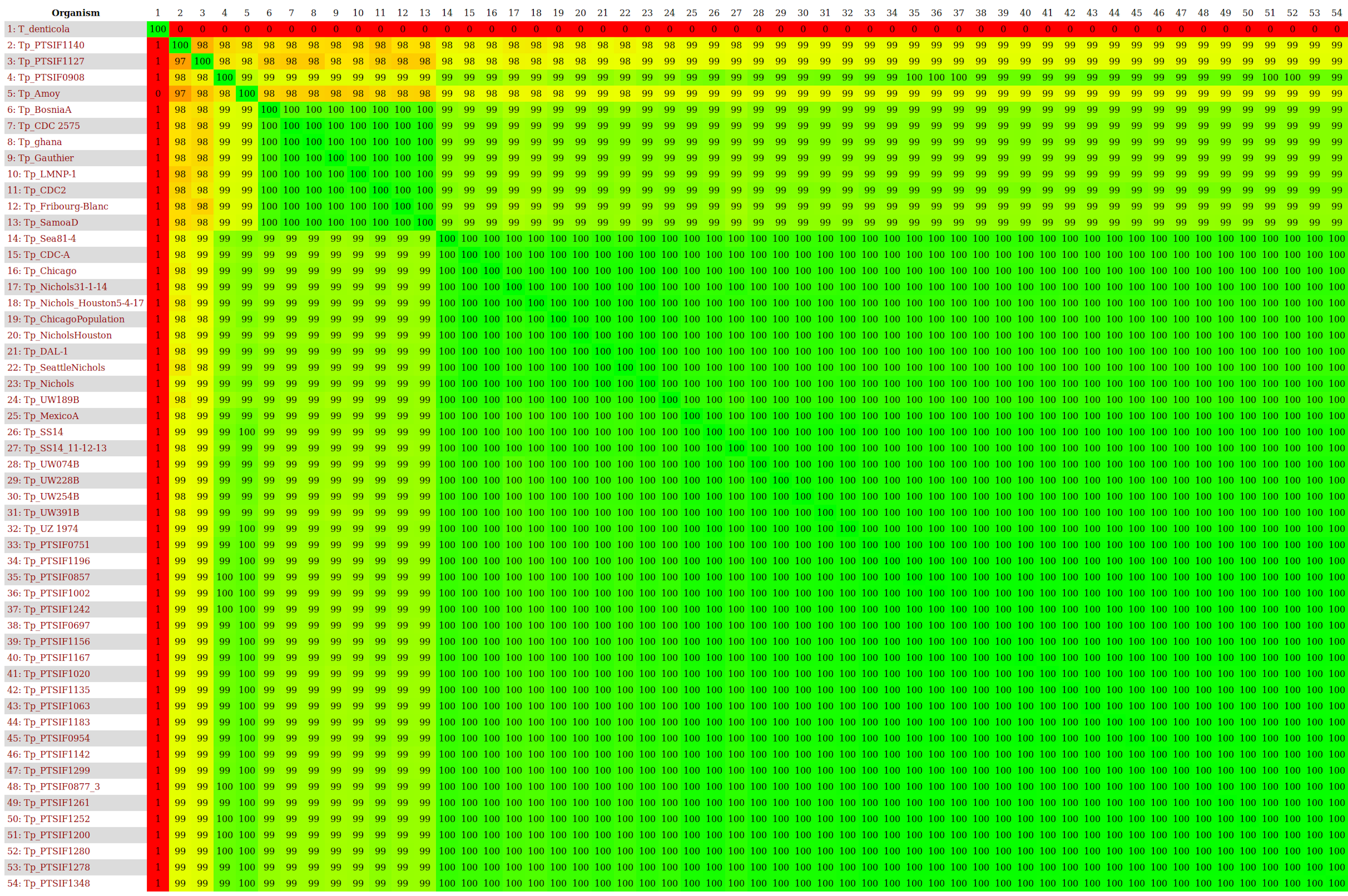
**
